# Paraneoplastic frosted branch angiitis as first sign of relapsed Hodgkin lymphoma

**DOI:** 10.1002/ccr3.1778

**Published:** 2018-08-29

**Authors:** Muhamad Alhaj Moustafa, Eric L. Crowell, Sherif Elmahdy, Vera Malkovska, Ashvini K. Reddy

**Affiliations:** ^1^ Department of Internal Medicine MedStar Georgetown University Washington Hospital Center Washington District of Columbia; ^2^ Division of Ocular Immunology Wilmer Eye Institute Johns Hopkins University Baltimore Maryland; ^3^ Division of Hematology and Oncology Department of Internal Medicine Cancer Institute MedStar Washington Hospital Center Washington District of Columbia

**Keywords:** blurry vision, frosted branch angiitis, Hodgkin lymphoma, paraneoplastic, retinal vasculitis

## Abstract

Frosted branch angiitis (FBA) is a rare form of retinal vasculitis with typical perivascular edema taking the shape of frost on a tree branch. It was reported only twice as the initial presentation of Hodgkin lymphoma (HL). Here, we present the first case of paraneoplastic FBA as the initial sign of HL relapse in an elderly female.

## 1 INTRODUCTION

Frosted branch retinal angiitis (FBA) is a descriptive term for a rare ophthalmologic disease with severe sheathing of the retinal vessels. This diffuse retinal periphlebitis was first described in Japan by Ito et al^1^ in 1976. Fewer than 90 cases of FBA have been reported since. FBA usually affects both eyes of young healthy patients.^2^ It is important to differentiate between primary FBA and secondary FBA which is associated with infectious, inflammatory, or neoplastic disorders. The identification of underlying causes has important implications for therapy. Reported conditions that can present with secondary FBA include herpes simplex virus retinitis,^3^ varicella zoster virus retinitis,^4^ cytomegalovirus retinitis,^5^ toxoplasmosis,^6^ tuberculosis,^7^ familial Mediterranean fever,^8^ antiphospholipid antibody syndrome,^9^ systemic lupus erythematosus,^10^ syphilis,^11^ HIV infection,^12^ Behçet's disease,^13^ Hodgkin lymphoma (HL),^14^, ^15^ and direct ocular invasion by malignant cells.^16^, ^17^

In primary FBA, the cause of perivascular infiltrates is not identified.^2^ Secondary FBA is either caused by direct involvement of the retina by viruses, bacteria, and malignancies or by the immune response to these disorders.^2^ Most patients respond to corticosteroids or to treatment of the underlying cause while few cases have recovered after symptomatic therapy with nonsteroidal anti‐inflammatory drugs.^18^, ^19^, ^20^, ^21^, ^22^ Most patients present with acute to sub‐acute visual loss, floaters, or photopsia. The visual acuity may be significantly reduced but can recover rapidly. Permanent visual damage is reported in 10% of the cases.^2^

Herein, we describe the first case of paraneoplastic FBA as the initial presentation of HL relapsing in an elderly female after 13 years of remission. In this case, the patient suffered from permanent visual changes which were refractory to treatment and persisted after complete remission of HL was achieved.

## 2 CASE DESCRIPTION

**Figure 1:**
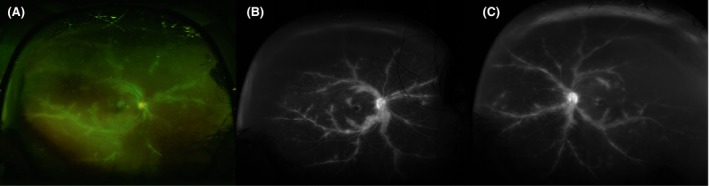
A, Optos fundus photograph of the right eye with frosted branch pattern periphlebitis. B, Fluorescein angiography of the right eye showing diffuse vasculitis, angiographic edema, and mild disc leakage. C, Fluorescein angiography of the left eye showing diffuse vasculitis, angiographic edema, and mild disc leakage

A 71‐year‐old African American female with a history of hypertension was diagnosed with stage IIa classical HL in 2003. She underwent four cycles of adriamycin, bleomycin, vinblastine, and dacarbazine (ABVD) achieving a complete remission (CR). In 2016, she developed progressive bilateral patchy visual loss over 4 months prior to seeking medical attention. She was seen by an ophthalmologist (AKR) who diagnosed bilateral FBA (Figure 1). Based on the ophthalmologic findings, patient was evaluated for HL relapse. She was otherwise asymptomatic and has gained 1.4 kg over the past year. Full blood count and chemistry profile were unchanged. Testing for other causes of FBA was negative, including fluorescent treponemal antigen absorption (FTA‐ABS), T‐spot, angiotensis‐1‐coverting enzyme, muramidase‐lysozyme, Antineutrophil cytoplasmic antibodies (ANCA), and toxoplasma IgG and IgM antibodies. Testing from an ocular fluid sample for viral causes was deferred by the patient.

Computed tomography (CT) scan of the chest, abdomen, and pelvis showed a mildly prominent left supraclavicular lymph node as well as enlarged nodes at the right iliac chain and right iliac fossa. Positron emission tomography (PET) scan showed diffuse involvement of the left supraclavicular, bilateral iliac chain, and retroperitoneal lymph nodes with maximum standardized uptake values (SUV‐max) of 10.7. There were right paracolic soft tissue tumor implants with SUV‐max of 5.8, and metabolically active sclerotic lesions in the left iliac bone with SUV‐max of 3.4. A right external iliac node biopsy confirmed classical HL with the same histological and immunohistochemical findings as the biopsy at presentation. Reed‐Sternberg cells were positive by immunohistochemistry for CD15 and CD30, and negative for CD45, CD20, CD3, EBER, and AE1/AE3. Flow cytometry showed no immunophenotypic evidence of monoclonal B lymphocytes or immune‐phenotypically abnormal T lymphocytes.

**Figure 2:**
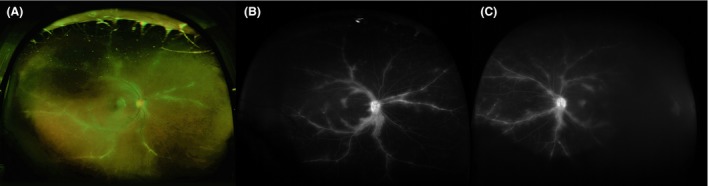
A, Optos fundus photograph of the right eye with frosted branch pattern periphlebitis with improvements. B, Fluorescein angiography of the right eye showing diffuse vasculitis with minimal improvements in angiographic edema and disc leakage. C, Fluorescein angiography of the left eye showing diffuse vasculitis with continued angiographic edema and disc leakage

Patient started intravenous therapy for HL with brentuximab vedotin and intraocular injections of bevacizumab 1.25 mg/0.05 mL monthly for 4 months. After two cycles of brentuximab, the patient achieved a CR by PET/CT scan. Because of the long first remission, patient's age, and preference, it was decided to continue brentuximab and not proceed with autologous stem cell transplant. Her vision improved from 20/30 to 20/25 OD (right eye) and worsened from 20/50 to 20/65 OS (left eye) over the course of 6 months after the beginning of HL treatment. The lack of improvement in the left eye was secondary to a choroidal neovascular membrane which developed subfoveally. On follow‐up imaging with fluorescein angiography her periphlebitis minimally improved in both eyes and she had resolution of her angiographic edema in the right eye, but no improvement in the left eye. (Figure 2).

## 3 DISCUSSION

Classical HL is a B‐cell lymphoprolifrative malignancy which usually presents as painless lymphadenopathy with or without constitutional symptoms; fevers, chills, drenching night sweats, and weight loss. Early ocular manifestations are extremely rare. Bilateral retinal periphlebitis as the initial presentation of HL was first described by Barr and Joondeph in 1983. Another case of FBA in HL was described in Belgium in 2009 by Hua et al. In the first case, systemic radiation therapy resulted in resolution of the ocular manifestations. In the second case, FBA resolved completely after 24 days of systemic steroids.^14^, ^15^ In both cases, it was suggested that FBA was a paraneoplastic phenomenon without direct malignant ocular invasion. In contrast, two cases of direct ocular invasion by primary central nervous system lymphoma and acute lymphoblastic leukemia, respectively, were described.^16^, ^17^ This has been described by Kleiner in 1997 as “frosted branch‐like appearance.”^23^

Our case is unique because it heralds a relapse of HL before any other signs or symptoms. Patient's advanced age is also unusual because FBA is a disease of young patients.^2^ The oldest reported patient with FBA was a 42‐year‐old female.^24^ FBA was the only warning sign for a relapsed HL after 13 years in remission in an otherwise asymptomatic female. Moreover, while most published cases of secondary FBA were acute to sub‐acute in presentation and resolved with therapy of the underlying disease, our patient's course was chronic and persisted. She presented 4 months after her vision started to deteriorate. This late presentation might explain the slow and incomplete recovery of her vision which could now be due to permanent changes.

Frosted branch angiitis represents a new entity on the list of paraneoplastic diseases that may occur in the context of HL. This report emphasizes the importance of searching for underlying lymphoma in patients presenting with this uncommon disorder.

## CONFLICT OF INTEREST

None declared.

## AUTHORSHIP

MA: wrote the manuscript. ELC and SE: critically reviewed the manuscript and helped with figures. VM and ARK: assisted in the medical management of the patient and critically reviewed this manuscript.
